# Multidisciplinary Approach in Atrial Fibrillation: As Good as Gold

**DOI:** 10.3390/jcm13164621

**Published:** 2024-08-07

**Authors:** Fabiana Lucà, Maurizio Giuseppe Abrignani, Fabrizio Oliva, Maria Laura Canale, Iris Parrini, Adriano Murrone, Carmelo Massimiliano Rao, Martina Nesti, Stefano Cornara, Irene Di Matteo, Michela Barisone, Simona Giubilato, Roberto Ceravolo, Carlo Pignalberi, Giovanna Geraci, Carmine Riccio, Sandro Gelsomino, Furio Colivicchi, Massimo Grimaldi, Michele Massimo Gulizia

**Affiliations:** 1 Cardiology Department, Grande Ospedale Metropolitano, GOM, AO Bianchi Melacrino Morelli, 89129 Reggio Calabria, Italy; massimo.rao@libero.it; 2 Operative Unit of Cardiology, P. Borsellino Hospital, 91025 Marsala, Italy; maur.abri60@gmail.com; 3 Cardiology Unit, ASST Grande Ospedale Metropolitano Niguarda, 20162 Milano, Italy; fabri.oliva@gmail.com (F.O.); dimatteoirene@hotmail.it (I.D.M.); 4 Division of Cardiology, Azienda USL Toscana Nord-Ovest, Versilia Hospital, 55049 Lido di Camaiore, Italy; marialauracanale@icloud.com; 5 Division of Cardiology, Mauriziano Hospital, 10128 Turin, Italy; irisparrini@libero.it; 6 Cardiology-ICU Department, Ospedali di Città di Castello e di Gubbio-Gualdo Tadino, AUSL Umbria 1, Via Guerriero Guerra, 06127 Perugia, Italy; adriano.murrone@gmail.com; 7 Division of Cardiology Fondazione Toscana G. Monasterio, 56124 Pisa, Italy; martina.nesti20@gmail.com; 8 Department of Translational Medicine, University of Piemonte Orientale, Via P. Solaroli, 17, 28100 Novara, Italy; stefano.cornara@gmail.com; 9 Cardiology Department, Cannizzaro Hospital, 95126 Catania, Italy; 10 Arrhytmia Unit, Division of Cardiology, Ospedale San Paolo, Azienda Sanitaria Locale 2, 17100 Savona, Italy; simogiub@hotmail.com; 11 Cardiology Unit, Giovanni Paolo II Hospital, 97100 Lamezia, Italy; roberto_ceravolo@yahoo.it; 12 Clinical and Rehabilitation Cardiology Department, San Filippo Neri Hospital, ASL Roma 1, 00135 Roma, Italy; carlo.pignalberi@aslroma1.it (C.P.); furio.colivicchi@gmail.com (F.C.); 13 Cardiology Division, Sant’Antonio Abate, ASP Trapani, 91100 Erice, Italy; giovannageraci@hotmail.com; 14 Cardiovascular Department, Sant’Anna e San Sebastiano Hospital, 81100 Caserta, Italy; carminericcio8@gmail.com; 15 Cardiothoracic Department, Maastricht University Hospital, 6229 HX Maastricht, The Netherlands; sandro.gelsomino@gmail.com; 16 Department of Cardiology, General Regional Hospital “F. Miulli”, 70021 Bari, Italy; m.grimaldi@miulli.it; 17 Cardiology Department, Garibaldi Nesima Hospital, 95122 Catania, Italy; michele.gulizia60@gmail.com

**Keywords:** multidisciplinary team, multi-integrated approach, atrial fibrillation

## Abstract

Atrial fibrillation (AF) represents the most common sustained arrhythmia necessitating dual focus: acute complication management and sustained longitudinal oversight to modulate disease progression and ensure comprehensive patient care over time. AF is a multifaceted disorder; due to such a great number of potential exacerbating conditions, a multidisciplinary team (MDT) should manage AF patients by cooperating with a cardiologist. Effective management of AF patients necessitates the implementation of a well-coordinated and tailored care pathway aimed at delivering optimized treatment through collaboration among various healthcare professionals. Management of AF should be carefully evaluated and mutually agreed upon in consultation with healthcare providers. It is crucial to recognize that treatment may evolve due to the emergence of new risk factors, symptoms, disease progression, and advancements in treatment modalities. In the context of multidisciplinary AF teams, a coordinated approach involves assembling a diverse team tailored to meet individual patients’ unique needs based on local services’ availability.

## 1. Introduction

Atrial fibrillation (AF) is considered the most common sustained arrhythmia, with its incidence and prevalence on the rise worldwide. This escalating burden stems from a multitude of factors, including the aging population, the surge in obesity rates, improved detection methods, and enhanced survival rates among individuals with AF and other cardiovascular diseases (CVDs).

Several studies have indicated that a considerable proportion, approximately one-quarter to one-fifth, of AF patients are admitted to emergency departments annually [1,2]. These emergency admissions are primarily attributable to symptomatic AF. However, they also underscore the presence of additional cardiovascular (CV) or non-cardiovascular (CV) comorbidities among AF patients, precipitating unplanned hospital visits.

Upon identifying the initial episode of AF, physicians must conduct a comprehensive evaluation to detect potential comorbidities. Subsequently, tailored treatment strategies for each comorbidity should be promptly initiated [3].

Integrated care for AF patients can be defined as a patient-centric approach encompassing all individuals affected by AF while concurrently establishing an efficient framework capable of delivering comprehensive care across all essential treatment domains. It is crucial to note that integrated care cannot be solely administered by a single healthcare provider but necessitates stringent collaboration among primary, secondary, and tertiary care settings [4].

In this context, the adoption of multidisciplinary teams (MDTs) is a pivotal strategy for delivering comprehensive and holistic care [5].

**Figure 1:**
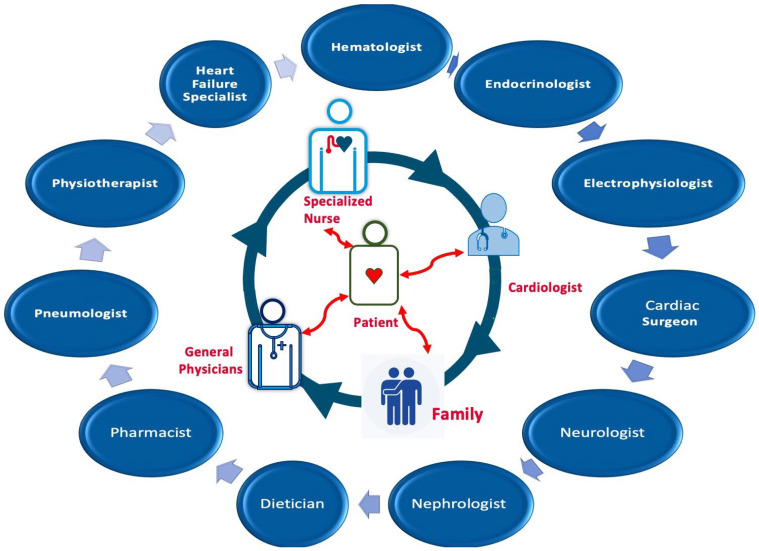
Multidisciplinary team for management.

The patient occupies a central role within the MDTs and is actively involved in daily decision making alongside other team members, including family, nurses, and specialist physicians (Figure 1). 

The initial management of AF patients primarily involves immediate or straightforward tests, typically conducted by acute or general doctors and specialized nurses. First-line treatment encompasses anticoagulation, antihypertensive therapy, lifestyle recommendations, and the critical rate or rhythm control decision, often in consultation with a dedicated cardiologist. Providing AF treatment knowledge to patients and their caregivers is a key component and predictor of adherence to drug treatments for AF [3,6]. The latest guidelines [3] delineate a significant paradigmatic shift, emphasizing the centrality of patient-oriented care in medical practice, wherein the focus pivots toward the individual patient rather than solely managing the disease entity, such as AF, and its acute complications. 

While most healthcare systems prioritize the acute management of AF, a structured approach to follow-up is indispensable for monitoring and addressing its multifaceted dimensions [7]. 

However, AF management is complicated and scarcely adheres to international recommendations in clinical practice due to the persistence of controversial issues, including treatment of comorbidities and pharmacological and interventional strategies. The aim of this review is to address the most controversial scenarios and provide pragmatic suggestions related to a multidisciplinary approach to AF.

We outline the primary comorbidities associated with AF, viewed through the lens of multidisciplinary care. We emphasize the critical role of teamwork in diagnosing and treating the multifaceted aspects of this complex arrhythmic condition.

## 2. Multidisciplinary Assessment of AF

AF is an arrhythmic disorder that necessitates dual attention: immediate management of acute complications and ongoing surveillance to modify the disease trajectory and ensure sustained patient welfare over time. Within the broad spectrum of AF patients, there exist diverse symptoms, potential complications, and concurrent diseases, ranging from arterial hypertension (AH) to obesity.

Numerous aspects of AF management necessitate specialized expertise for optimal handling. Substantial care gaps in AF management have been reported [8]. These gaps encompass pivotal decisions, such as identifying the candidates for anticoagulation, timing cardioversion procedures, determining the transition from rate to rhythm control strategies, and accurately referring patients for catheter ablation (CA) interventions [8].

Establishing a dedicated clinic under the supervision of a physician holds promise for improving these challenges. It would lead to an evidence-based strategy, delineate novel therapeutic interventions, and enhance the understanding of current AF management paradigms.

In light of this, MDTs’ integrated AF approach is increasingly desirable and necessary [9]. Prior research has suggested that a collaborative model involving specialists and nurses in AF clinics is highly beneficial, resulting in a noteworthy reduction in emergency department visits and hospitalizations and improved patient survival rates. Moreover, the importance of addressing acute complications secondary to AF has been recently highlighted [10]. 

**Figure 2:**
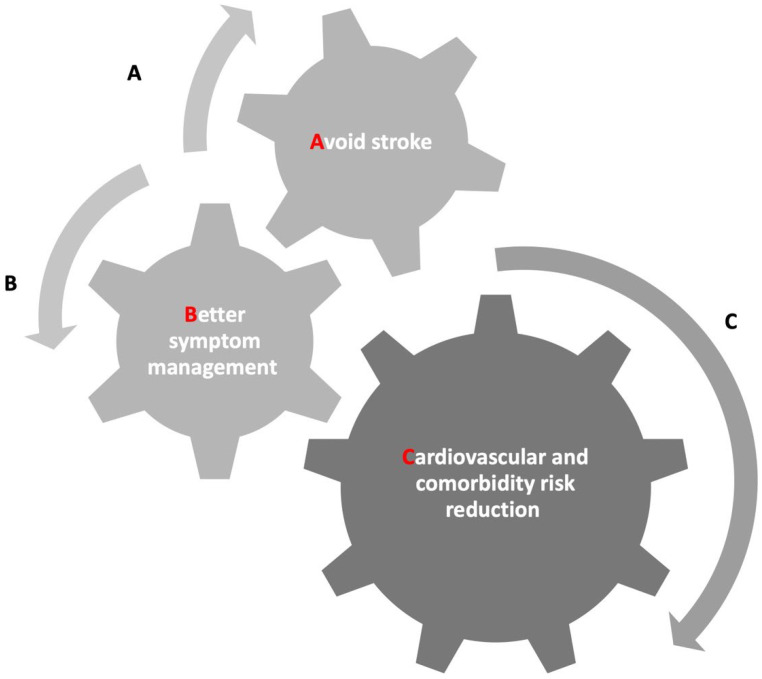
(**A**–**C**) Atrial fibrillation better care (ABC) pathway.

In this sense, the ESC guidelines advocate for the adoption of the ABC algorithm (“A”: Anticoagulants to prevent stroke; “B”: Symptom management optimization; “C”: Comprehensive management of cardiovascular health and comorbidities) [9] as a strategy to mitigate the risk of adverse clinical outcomes associated with AF (see Figure 2A–C and Table 1).

While many healthcare systems predominantly emphasize acute AF management, a structured approach to long-term monitoring and intervention, including cerebrovascular event prevention, is indispensable for comprehensively addressing the multifaceted dimensions of AF. Regarding the “A” component of the algorithm (see Figure 2A), it is important to note that cerebrovascular event prevention represents the main goal in AF management. Indeed, each individual risk factor raises the likelihood of a stroke or mortality [20]. Therefore, adequate stroke prevention must be performed assuming an adequate OAC therapy. However, the predictive value of the risk scores used to stratify ischemic risk remains poor for identifying patients at a higher risk of worse outcomes. An effective cerebrovascular accident strategy may be obtained using direct oral anticoagulants (DOACs) or vitamin K antagonists (VKAs) in those patients who are not eligible for DOACs, maintaining time in the therapeutic range (TTR) >65–70% [21]. On the contrary, it is essential to identify patients with a low CHA2DS2-VASc score (0 for males and 1 for females), characterized by a low risk of thromboembolic events (<1% per year), who do not require any oral anticoagulants (OACs). Moreover, the adherence rates to OACs therapy in real-world settings exhibit considerable variability across different studies [22,23]. A bleeding risk factor evaluation should be performed before initiating therapy, focusing on potentially modifiable risk factors to improve hemorrhagic risk stratification. Moreover, a score for evaluating the bleeding risk, the HAS-BLED score, should be used for identifying patients at a high hemorrhagic risk [21].

DOACs should be preferred, according to the latest guidelines [24,25,26]. 

The next focus is managing symptoms and choosing between rate or rhythm control (see Figure 2B). The symptoms may lead to the initial strategy. Moreover, physicians should decide how to manage AF, tailoring the strategy to patients’ characteristics [27]. 

The rhythm control approach aims to relieve symptoms and improve functional status. In large randomized controlled trials (RCTs) and meta-analyses [28,29,30], rhythm control has been compared to the rate control approach in AF, revealing no significant difference in the risk of all-cause mortality despite the findings seeming to favor the rhythm control strategy slightly [3].

In the AFFIRM [30], an all-cause mortality reduction trend in the rhythm control group has been reported without differences in cardiac and arrhythmic mortality and stroke. Nonetheless, it has been reported that the potential benefits of antiarrhythmic drugs (AADs) may have been counterbalanced by their side effects [31].

In RACE [29,32], a trend of lower risk of CV mortality, HF hospitalizations, and thromboembolic and hemorrhagic complications has been reported in the rhythm control group. However, the rhythm control strategy has been associated with better outcomes in patients with a recent diagnosis (within one year) of AF [33,34,35,36,37].

According to more recent data, in patients who received a recent AF diagnosis (within 12 months), the risk of adverse CV events seems to be lower with the rhythm control approach than with standard care [33,36].

In EAST-AFNET4 [33], patients undergoing early rhythm control had lower CV mortality, stroke, HF hospitalization, and acute coronary syndrome (ACS).

When a rate control strategy is adopted, beta-blockers (BBs) are the most common choice [3], with a favorable safety and efficacy profile. Other choices include non-dihydropyridine calcium-channel blockers (CCBs), digoxin, and amiodarone, taking into account factors such as left ventricle (LV) function and the coexistence of other comorbidities. Non-dihydropyridine CCBs (diltiazem and verapamil) may be used in both acute and chronic settings and are frequently added to BBs, except for patients with HF and manifest pre-excitation. Digoxin may be used as a second choice in chronic patients with lower cardiac rates, especially in combination with BBs or non-dihydropyridine CCBs, with particular attention to blood levels. If verapamil is used, care should be taken in dosing digoxin levels because of the potential interaction, which raises digoxin levels. Therefore, according to the available evidence, the decision to pursue a rate control or rhythm control strategy is not straightforward. It should be based on a comprehensive evaluation specific to each patient, including a careful assessment of the symptoms, ejection fraction, and cardiac monitoring to evaluate the effectiveness of rate control and patient preference.

Moreover, in some cases, such as in elderly and frail patients, especially in those asymptomatic patients with preserved LV function [30,38], the rate control strategy may represent the most suitable approach, whereas in other cases, it may be considered as an intermediate step before reaching CA therapy. CA has been considered the treatment of choice in AF patients with paroxysmal forms and intolerance or refractoriness to at least one AAD [38,39,40]. Pulmonary vein antrum isolation (PVI) is performed by creating circumferential lesions around the right and the left pulmonary veins (PVs) of the antrum, using radio frequency (RF) energy or the cryoablation method [41].

Moreover, newer ablation systems, such as balloon-based laser endoscopic ablation, have been introduced with satisfying results in terms of safety and efficacy [42,43,44].

Notably, CA has been considered the first line of therapy in a specific subset of patients to interrupt anticoagulation and antiarrhythmic drugs. Findings from CABANA [45], which compared CA with AAD therapy, evidenced that all-cause mortality, disabling stroke, hemorrhagic events, and cardiac arrest were not significantly different in a five-year control period. In contrast, a significant reduction in CV mortality and hospitalization was achieved in the CA group. 

Therefore, accurate evaluation of which strategy best suits the patient’s characteristics assumes a pivotal role and focuses the therapeutic line on those characteristics.

The third point of the ABC algorithm consists of comorbidities management (see Figure 2C). Indeed, in light of the fact that AF commonly coexists with a complex burden of CV risk factors and comorbidities, including psychological disorders [46], AH, HF, diabetes mellitus (DM), obstructive sleep apnea syndrome (OSAS), obesity, and coronary artery disease (CAD), integrated management assumes a pivotal role in enhancing patient care [47]. 

**Figure 3:**
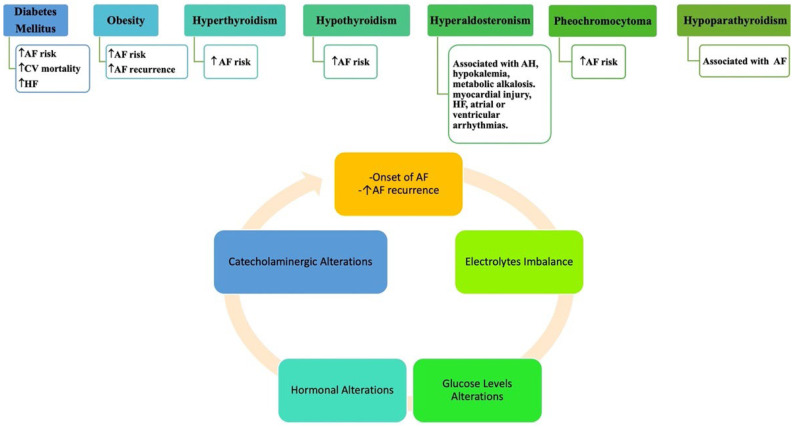
Association between endocrinological disorders and AF. AF and endocrine disorders are commonly associated. Diabetes mellitus (DM) has been associated with a heightened risk of AF. Obesity is linked to an elevated susceptibility to AF. A relationship between thyroid disorders and AF has also been well assessed. Pheochromocytoma, hyperaldosteronism, and hypoparathyroidism have also been associated with AF. Abb: AF: atrial fibrillation; AH: arterial hypertension; HF: heart failure. ↑: Increasing.

A correlation between AF and the most prevalent endocrine disorders has been shown [48] due to electrolytic and metabolic imbalances and hormone alterations that may create an environment conducive to the onset and recurrence of AF [49] (see Figure 3).

A comprehensive multi-specialistic approach involving patients, their families, nurses, general doctors, and other specialists is crucial for better management of comorbidities.

Education and counseling can enhance patient outcomes and adherence to treatment, increasing awareness and changing lifestyles [50].

However, adherence to the ABC algorithm remains suboptimal, with only one out of five patients adhering to it, according to a recent meta-analysis [51]. Furthermore, adherence to the ABC pathway tends to diminish as comorbidities increase. Several studies have highlighted this aspect of AF management [22]. It is important to underline that incorporating specialized nurses in AF follow-up has shown significant promise in various studies, with randomized observational data demonstrating improvements in hospitalization rates and even CV mortality. This improvement is attributed to enhanced adherence to therapy and adherence to the ABC algorithm [52,53]. To the best of our knowledge, the RACE 4 trial represents the initial randomized multicenter clinical trial to demonstrate the superiority of nurse-led care follow-up, albeit predominantly evident when conducted in experienced centers [54]. Another significant trial, the CARE-AF (ACTRN12616001109493) trial, was specifically designed to address the management of patients with chronic conditions and multiple morbidities in the context of AF. The outcomes of this trial are anticipated to provide further insights into the importance of adopting an integrated approach to AF management.

Studies show that nurse-led management, in collaboration with the MDTs, reduces CV hospitalization and improves patients’ quality of life with AF, suggesting that this innovative management approach should be implemented in clinical practice [55,56,57].

## 3. Stroke and Cognitive Impairments

AF raises the risk of stroke by five-fold, and strokes associated with AF are more severe, exhibiting higher rates of mortality and disability. Furthermore, stroke represents a significant risk factor and predictor for cognitive impairments (CIs) and dementia, affecting up to one-third of stroke survivors [20]. The risk of post-stroke CI and dementia seems to correlate with stroke severity, site, and recurrence [58].

**Figure 4:**
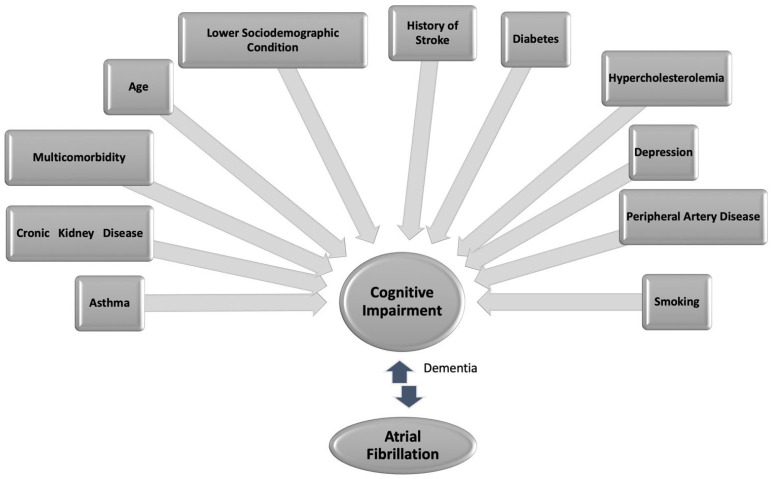
Association between cardiovascular risk factors, comorbidities, AF, and cognitive impairment. Cognitive impairment and AF share many risk factors and underlying comorbidities and correlate with the development of dementia in AF patients.

Currently, AF and CI constitute a major health problem worldwide, and their prevalence is expected to increase exponentially in the coming decades with population aging. Earlier research has demonstrated that patients with AF face a 2.5-fold higher risk of CI compared to those without a history of stroke and a 1.5-fold higher risk compared to patients without AF [59,60,61]. This correlation remains independent of common risk factors, such as aging, hypertension, and DM. Furthermore, it appears more pronounced among younger individuals than among the elderly [62,63]. Moreover, the temporal relationship, with AF preceding cognitive decline and the elevated risk of CI associated with increasing AF burden (time spent in AF), suggests a causal link between AF and CI (Figure 4).

The exact pathophysiological mechanisms by which patients with AF may develop CI and dementia are still not fully understood, but several hypotheses have been postulated. AF can lead to macro- and micro-thromboembolism through various mechanisms, resulting in a clinical stroke, silent stroke, or micro-infarcts. These occurrences can cause morphological changes in the brain, contributing to CI or dementia. Embolic-related micro-infarcts, even in the absence of a documented clinical stroke, increase the risk of dementia in patients with AF; thus, recent studies have observed a high burden of ischemic lesions in brain magnetic resonance imaging (MRI), which is presumed to cause progressive deterioration of cognitive function [64,65].

Other studies demonstrated that patients with both AF and silent cerebral infarction were more likely to have CI than those with AF alone, suggesting the pathogenetic role of macro- and micro-thromboembolism [66,67].

Another mechanism involved could be cerebral hypoperfusion. Indeed, AF can result in reduced cardiac output and cerebral blood flow. Due to decreased cardiac performance, the beat-to-beat variability may also induce impaired cerebral perfusion. 

The association persists despite typical risk factors like aging, hypertension, and DM. Interestingly, it seems more prominent among younger individuals than the elderly [68]. Moreover, the elevated occurrence of cerebrovascular ischemic events and dementia in AF patients who also have concurrent HF lends additional support to the cerebral hypoperfusion hypothesis. Cerebral micro-bleeds (CMBs) have been suggested as another possible trigger, particularly in patients on warfarin therapy with a high international normalized ratio (INR) [69]. Indeed, patients with CMBs seem to be more likely to develop stroke, particularly intracerebral hemorrhage (ICH) [70,71,72,73]. CMBs have been suggested to correlate with CHADS2 and CHADS2-VASC scores [74,75]. 

CMBs have been shown to consist of hemosiderin deposition due to minor bleeding caused by micro-angiopathy, frequently coexisting with cerebral amyloid angiopathy (CAA), arteriosclerosis, and AH [76]. Deep or lobar (cortical/subcortical) localizations have been reported depending on arteriosclerosis/AH or CAA as the underlying causes, respectively. A mixed distribution has also been described in patients with both CAA and AH or in those with an isolated HA. CMBs may be detected by cerebral MRI [77]. Moreover, a CMB prevalence of 21% has been described in the general population, which may reach >40% in patients older than 80 years, 35% in those with previous cerebrovascular ischemic events, and 20–80% in those with coexistent ICH history [78,79,80]. CMBs have been hypothesized to be an independent predictor of ICH. Moreover, an association between the number of CMBs and ICH risk has been proposed [81,82]. Furthermore, patients with multiple lobar CMBs are more likely to develop an ICH [83]. 

AF is linked to elevated levels of inflammatory biomarkers, including C-reactive protein, interleukin (IL)-2, IL-6, and IL-8, as well as tumor necrosis factor-alpha. This proinflammatory condition may promote prothrombotic states and exert direct inflammatory effects on the brain, contributing to cognitive dysfunction [84,85]. Interestingly, statin therapy seems to positively impact cognitive outcomes in patients with AF [86].

Genetic risk factors have undergone extensive research; however, their potential role as a link between AF and cognitive dysfunction remains uncertain. In a study by Rollo et al., an association was found between the PITX2 loci, particularly rs2200733, and dementia [87]. Nevertheless, further research is required to confirm these findings and to elucidate the role of genetic factors that might impact the development of cognitive dysfunction in individuals with AF.

Cerebral hypoperfusion and AF-related ischemic brain injury of cardioembolic origin may affect vascular beds and thereby reduce brain volume. Specifically, AF has been associated with predominant frontal lobe atrophy, and these findings have been correlated with impaired cognitive function [88]. Chronic cerebral hypoperfusion and the proinflammatory state in AF may facilitate amyloid deposits in brain tissue, potentially linking AF with Alzheimer’s disease. Neuropathological studies have demonstrated that senile plaque formation and amyloid angiopathy are increased in patients with a history of permanent AF compared with controls without AF [89].

If we consider a connection between dementia and AF, treatment with OACs could potentially mitigate the risk of cognitive decline in AF patients. Observational studies hint at a possible link between anticoagulant therapy, particularly when maintaining optimal TTR with warfarin, and a decreased rate of cognitive decline. This implies that embolism may play a role in cognitive impairment. However, the presence of potential confounding variables in these studies hampers the definitive establishment of a cause-and-effect relationship [69]. Moreover, subsequent studies and meta-analyses have failed to demonstrate a clear benefit of anticoagulation for cognitive decline in patients with AF [90,91,92].

Although previous studies have found no significant differences between DOACs and warfarin in the risk of new-onset dementia [93], DOACs provide more stable therapeutic levels, are equally effective in stroke prevention, and are associated with a lower risk of bleeding. Therefore, they may be a promising therapeutic strategy to reduce the risk of CI and dementia in patients with AF [94]. A recent Australian study analyzing data from 18,813 AF subjects followed up for an average of 3.7 years showed that the incidence of dementia was significantly lower in patients anticoagulated with DOACs than in non-anticoagulated patients [95]. Experimental studies in animal models show that, compared with warfarin, rivaroxaban treatment attenuated neuroinflammation, blood–brain barrier dysfunction, and beta-amyloid deposition and that dabigatran would have a positive effect on cognitive and psychological function, as well as on the pathogenesis of dementia, including Alzheimer’s disease [96].

Rhythm control, such as cardioversion or AF ablation, may improve cardiac output and cerebral perfusion. A recent meta-analysis of observational studies showed a potential reduction in dementia in AF patients treated with CA [97]. However, data concerning the impact of rate control in AF on the occurrence of CI and dementia are still not conclusive.

Additional large-scale prospective studies are required to investigate the strategies aimed at preventing dementia. These studies should also assess whether screening for undiagnosed AF, followed by targeted therapy, could potentially prevent or alleviate cognitive impairment and dementia.

## 4. Frailty and the Elderly

Frailty is an age-associated syndrome characterized by decreased physical strength, resilience, and functional capacity, making individuals more vulnerable to adverse health outcomes, disability, and dependency [98]. Its incidence has been reported to increase from 2.9% among individuals aged ≥65 to 25% among those aged ≥80. Additionally, women are more likely to exhibit frailty than men [98]. Frailty has been significantly associated with poor outcomes and mortality in patients with CVD, particularly in elderly patients [99,100]. Therefore, identifying this condition assumes a pivotal role in evaluating procedural risks and deciding on the most suitable management of CVD [101]. At the same time, the prevalence of AF increases exponentially with advancing age. In addition, elderly patients more commonly exhibit comorbidities, multiple chronic conditions, and frailty, all of which contribute to the worse outcomes of elderly AF patients compared to younger AF patients [102,103,104,105]. Importantly, in AF patients, frailty may significantly influence therapeutic strategy [106]. A frailty prevalence of 4.4–75.4% in AF patients has been reported. More importantly, frail patients with AF are also at a higher risk of developing thromboembolic events, exhibiting a greater incidence of CI and dementia, and experiencing a worse prognosis [107,108]. While it is well established that older age and frailty are correlated with an increased incidence of stroke and mortality among patients with AF, there exists evidence of a risk-treatment paradox. Interestingly, despite being at the highest risk of complications from AF, these patients are paradoxically less likely to receive treatment with OACs [109,110,111]. However, advanced age, frailty, comorbidities, and an increased risk of falls should not be considered absolute contraindications to OACs, as the expected benefit often outweighs the absolute risk of major and fatal bleeds. Multiple RCTs, meta-analyses, and large registries provide evidence to support the use of OACs in this age group, with DOACs seeming to have a more favorable overall benefit–risk profile than warfarin [112,113]. A recent systematic review demonstrated that warfarin was superior to aspirin and no antithrombotic therapy in the prevention of stroke/thromboembolism in elderly patients with AF. Still, it was associated with an increase in MB. On the other hand, DOACs were found to be superior to warfarin for stroke/thromboembolism prevention, with a reduced risk of major bleeding [114]. Additionally, data from the PREFER registry have supplied evidence of a net clinical benefit of OACs, encompassing DOACs, even in extremely elderly individuals residing in the community (aged ≥ 85 years) [115]. These findings further endorse the recommendation of DOACs as the preferred initial therapy for stroke prevention in elderly patients with AF [21]. However, it is crucial to acknowledge that some elderly AF patients may face an elevated risk of short-term all-cause mortality, which could potentially attenuate the indisputable benefit of DOACs. Unfortunately, no validated methods exist to identify elderly patients who may not derive a net clinical benefit from DOACs. Data supporting the superiority of DOACs in older and frail AF patients are scarce, consisting mainly of observational studies on the effect of frailty on bleeding outcomes [116]. Indeed, frail patients are vastly underrepresented in the RCTs, and only limited evidence from observational studies has been reported [117,118]. Therefore, establishing a better approach to optimal anticoagulant treatment is challenging. Whether DOACs are superior to VKAs in frail AF patients and the benefits of switching VKAs to DOACs remain under debate [119,120]. A nationwide study conducted in Korea in 2022 indicated that frail AF patients treated with DOACs had a generally lower risk of adverse outcomes compared to those treated with warfarin [121]. Additionally, a Medicare data analysis revealed that apixaban (but not dabigatran or rivaroxaban) was linked to fewer adverse events than warfarin in frail patients [121]. The FRAIL-AF study on 1330 AF frail older patients who switched to DOACs compared to those who continued with VKAs revealed associations with more hemorrhagic events without benefit in terms of reduction in ischemic complications [122]. In frail patients with non-valvular AF, rivaroxaban significantly reduced the hazard of developing stroke or systemic embolism without increasing the rate of serious bleeding compared with warfarin [123]. In the ARISTOTLE study, the efficacy and safety of apixaban remained consistent in older patients with greater morbidity and higher CHA2DS2-VASc scores compared to those with fewer complications [124]. Additionally, findings from the ENGAGE AF-TIMI 48 trial revealed that edoxaban is linked to an even more substantial absolute decrease in severe bleeding events and overall mortality when contrasted with warfarin in patients with AF who are at an elevated risk of falling [125]. The ELDERLY CARE Trial among 984 AF patients aged ≥80, not eligible for OAC at standard doses, showed that edoxaban (once-daily 15 mg dose) was superior to placebo in thromboembolic prevention without a significant increase in major bleeding incidence compared to placebo [126]. In a recent meta-analysis encompassing four studies and involving 835,520 patients, it was revealed that among individuals with AF and frailty, DOACs demonstrate superior efficacy and safety in comparison to warfarin. This superiority was evident in reducing the risk of stroke (both ischemic and hemorrhagic), all-cause mortality, major bleeding, and intracranial hemorrhage. However, no significant difference was observed in the incidence of gastrointestinal bleeding [112].

Accordingly, the current guidelines and consensus documents recommend considering anticoagulation even in older and frail patients after a careful case-by-case evaluation of potential risks and benefits [3,127]. In this regard, multidisciplinary management, including a geriatric assessment, is strongly recommended. In particular, screening and assessment for frailty should be systematically performed and integrated into the care management of these patients to mitigate the challenges surrounding anticoagulant treatment [128,129]. Complex elderly patients with comorbidities and frailty may exhibit reduced adherence to pharmacological treatment and an increased susceptibility to adverse drug events. Consequently, this patient population should require specific interventions, including careful clinical surveillance, periodic re-evaluation of co-medications, and strategies to minimize the risk of falls and bleeding. Although rate control is the preferred strategy in elderly patients with AF, there is insufficient evidence to inform the choice between rate and rhythm control in the elderly [130,131]. If indicated, rhythm control strategies such as cardioversion, pacemaker implantation, and CA for AF should be performed without age discrimination. CA of AF may be an effective and safe option in selected elderly subjects, with success percentages comparable to those of younger patients and acceptable complication rates [132,133].

## 5. Heart Failure

AF and HF are two of the most common CVDs, frequently coexisting and interconnected. A widely adopted concept is that AF leads to HF and vice versa, as they share common pathophysiological mechanisms and risk factors. 

AF involves the loss of atrial systole by reducing left ventricular (LV) filling, which reduces cardiac output by up to 25%. Additionally, rhythm irregularity and an excessively high heart rate during AF may increase the likelihood of developing a reversible form of LV cardiomyopathy—the so-called tachycardiomyopathy. On the other hand, HF can induce AF through several mechanisms, such as atrial pressure enlargement and overload, maladaptive gene expression, altered myocardial conduction, and structural cardiac remodeling [134,135]. Moreover, these conditions have a negative prognostic impact on each other [136,137,138,139], raising challenges in therapeutic management.

The current guidelines recommend a holistic approach to the management of these patients, including optimal medical therapy for HF, identification and treatment of potentially reversible causes or triggers of AF, such as electrolyte disturbances, hyperthyroidism, uncontrolled hypertension, and infection, anticoagulation for stroke prevention, and not least, rate or rhythm control [3,140].

Patients with HF and AF represent a population with a particularly high risk of thromboembolism [140,141,142]. 

Although the optimal heart rate target in patients with AF and HF is unclear, a heart rate < 110 bpm is generally recommended. The pharmacological approach to rate control differs between patients with heart failure with preserved ejection fraction (HFpEF) and reduced ejection fraction (HFrEF) [143]. In HFpEF, the possible therapeutic alternatives include beta-blockers (BBs), calcium-channel blockers (CCBs) such as diltiazem and verapamil, and digoxin. On the other hand, in patients with HFrEF, the recommended drugs are BBs. Moreover, digoxin should be considered on top of BBs when the heart rate remains high, despite the maximum tolerated dose of BBS, or when BBs are contraindicated. In addition, amiodarone i.v. should be used for rate control only in patients with hemodynamic instability. Importantly, amiodarone is known to be a P-glycoprotein system inhibitor, resulting in increased serum levels of digoxin and posing a risk of intoxication.

Urgent electrical cardioversion (CVE) is strongly recommended in the setting of acute worsening HF with concomitant AF and rapid ventricular rate [3,144]. In contrast, amiodarone is the drug of choice in case of pharmacological cardioversion. Maintaining SR is particularly challenging in the non-acute setting, as the majority of antiarrhythmic drugs (AADs) are contraindicated or poorly tolerated. The only effective drug is amiodarone; however, its use poses challenges due to its side effects, particularly in young patients [3,145].

In recent years, several randomized controlled trials (RCTs) and meta-analyses have demonstrated the potential advantages of CA in patients with AF and concurrent HF compared to pharmacological treatment [146,147,148,149,150]. Therefore, according to the current guidelines, catheter ablation (CA) should be considered for patients with HFrEF and deteriorating symptoms despite OMT in order to improve survival and reduce unplanned hospitalization (Class IIa). The so-called “ablate and pace strategy”, consisting of ablation of the atrioventricular (AV) node with permanent pacemaker implantation, should be considered in patients with permanent AF and pharmacologically refractory rapid ventricular response, which may induce tachycardia-related cardiomyopathy. Additionally, this option should be considered when a cardiac resynchronization therapy (CRT) is indicated in order to increase the biventricular pacing percentage [3,140].

## 6. Chronic Obstructive Pulmonary Disease (COPD) 

Chronic obstructive pulmonary disease (COPD) is a major global health issue, representing the fourth cause of death worldwide [151], with a prevalence of 14.3% and 7.6% among men and women aged > 30, respectively [152]. CVD and COPD frequently coexist [153,154] due to the occurrence of several common risk factors. Nonetheless, CVD and COPD have been shown to be independently associated, negatively affecting the prognosis and mortality [155].

An increased AF vulnerability under severe hypoxemia has been reported in COPD patients [156]. 

**Figure 5:**
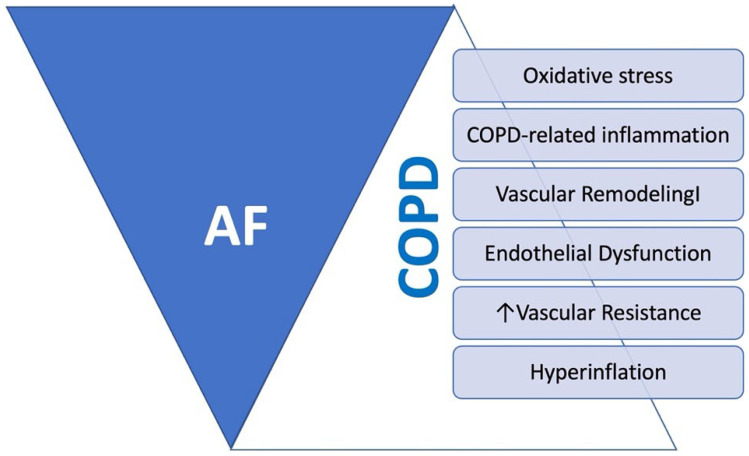
Mechanisms of COPD exacerbating AF onset and recurrence.

Hypercapnia is also linked to AF occurrence in COPD, significantly increasing atrial refractoriness and slowing atrial conduction [156]. While normalizing CO_2_ levels rapidly restores refractoriness, conduction slowing persists, potentially creating a substrate for AF following correction [156]. Additionally, hypercapnia and hypoxemia induce pulmonary arteriolar constriction, leading to pulmonary arterial and right ventricular hypertension, which may cause arrhythmias through right atrial dilation and increased transmural pressure on endocardial vessels [157]. AF may be triggered by higher arterial partial pressure of carbon dioxide (PaCO_2_) and pulmonary artery systolic pressure [158] during COPD exacerbations. Recent studies suggest prolonged and altered atrial depolarization in COPD patients. Right atrial (RA) electromechanical delay is significantly prolonged and negatively correlated with forced expiratory volume in the first second (FEV1), with COPD patients showing extended atrial depolarization duration [159] (Figure 5).

Acar et al. found that atrial electromechanical delay prolongation measured from the lateral tricuspid annulus is independently related to the forced vital capacity (FEV)1/FVC ratio [160]. Increased P-wave dispersion (PWD)—the difference between maximum and minimum P-wave duration—is an independent risk factor for AF development, more pronounced in the acute phase and in patients with frequent exacerbations, indicating its potential as a target for predicting, preventing, and treating acute COPD exacerbations [161,162]. P-wave duration and PQ interval are also risk factors for AF, with a strong correlation between P-wave duration and PQ interval > 150 ms [163]. However, little value in using P-wave signal-averaged electrocardiography (SAECG) and atrial late potential detection for arrhythmic risk assessment in acute COPD exacerbations has been suggested [163]. Furthermore, COPD is associated with LV diastolic dysfunction [164,165,166], which correlates with disease severity and may be another mechanism for AF initiation and perpetuation [164,165,166]. Right ventricular (RV) systolic dysfunction and elevated systolic pulmonary artery pressure are independently linked to the RA volume index in pulmonary hypertension (PH) patients due to COPD, potentially promoting AF [164]. Hemodynamic overload or stretching of the RA from PH may increase the prevalence of non-pulmonary vein foci from the RA in COPD patients [167]. Oxidative stress and inflammation are key pathogenetic mechanisms in COPD [168] and are also implicated in AF initiation and perpetuation [168,169]. Therefore, it is plausible that COPD-related oxidative and inflammatory responses may promote AF development. 

Importantly, on the one hand, identifying the incidence of AF in patients with COPD is crucial; on the other hand, COPD has been reported to predict AF progression and worse outcomes independently [170]. Indeed, the occurrence of COPD in AF patients has been associated with an increased CV and all-cause mortality and with a higher thromboembolic and hemorrhagic risk [171].

Conversely, the absence of COPD has been considered a predictor of successful CVE and maintenance of sinus rhythm in one year [172]. 

## 7. Obstructive Sleep Apnea (OSAS)

**Figure 6:**
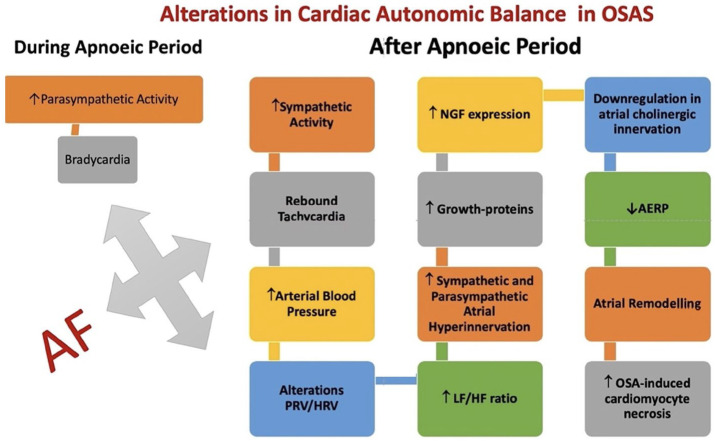
Relationship between obstructive sleep apnea and atrial fibrillation. Abb: HRV: heart rate variability; PVR: pulse rate variability; LF/HF: low-to-high frequency ratio; AERP: atrial effective refractory period; NGF: nerve growth factor. ↑: increase; ↓: decrease.

OSAS is a prevalent chronic condition affecting approximately 2–4% of adults, with a higher prevalence in older men [173,174,175]. This disorder is marked by recurrent episodes where the upper airway either partially or fully collapses during sleep, leading to reduced or halted airflow [176]. OSAS has been linked to the development of various CVDs, such as arrhythmias, AH, HF, and stroke [175,177]. There is a noted association between AF and OSA, and it has been determined that both conditions share numerous common risk factors, including sex, age, obesity, DM, smoking, and Helicobacter pylori infection [178]. Several mechanisms may contribute to the onset and progression of AF in individuals with OSAS [179]. During the acute phase, patients experience hypoxia, hypercapnia, changes in intrathoracic pressure, increased sympathetic activity, and autonomic dysfunction [179] (Figure 6). These repeated events can lead to structural and functional changes in the atria, including atrial remodeling and fibrosis. Continuous positive airway pressure (CPAP) therapy has been shown to reduce AF burden, regardless of the rhythm control strategy, including antiarrhythmic drugs, direct current cardioversion, or CA [180,181]. Moreover, CPAP treatment has been associated with a lower likelihood of developing permanent AF compared to not using CPAP [175].

Patients with AF and OSAS face a heightened risk of AF recurrence following CA and show a higher prevalence of non-pulmonary vein triggers [182,183]. A relationship between OSA and AF substrates characterized by low left atrial voltage has been reported using high-density mapping. Their findings demonstrated a dose-dependent association between OSA severity and increased conduction heterogeneity, voltage heterogeneity, and the number of low-voltage areas throughout the left atrium (LA). The apnea–hypopnea index (AHI) correlated with lower overall left atrial voltage but not with reduced conduction velocity. For patients with paroxysmal AF and OSA, discrete low-voltage zones paired with slow conduction velocity were identified as the atrial substrate. Conversely, patients with persistent AF exhibited a more widespread low-voltage pattern across the LA.

## 8. Chronic Liver Diseases 

Liver function is crucial for maintaining homeostasis and preventing excessive blood clotting. Individuals with advanced chronic liver disease (CLD) face a heightened risk of both clotting and bleeding. This is due to thrombocytopenia and diminished synthesis of fibrinogen and other clotting factors (such as II, V, VII, IX, X, XI, and XII). Additionally, low levels of C protein, antithrombin, and plasminogen, coupled with increased von Willebrand factor activity, further elevate the risk of thrombosis. AF has been linked with CLD, including forms caused by metabolic disorders, such as non-alcoholic fatty liver disease (NAFLD), as well as non-metabolic factors like alcohol consumption or viral hepatitis [184]. NAFLD has emerged as the leading CLD worldwide, affecting around 25% of the adult population [185]. NAFLD is linked to an approximately two-fold increase in the incidence of AF in the general population, which is up to six-fold in those with DM [186]. Furthermore, NAFLD elevates the risk of AF in middle-aged and elderly individuals [187]. A strong linear relationship between NAFLD and AF risk has been reported, with a 2.1-fold higher likelihood of an AF diagnosis, regardless of other risk factors [187]. 

Shared risk factors between AF and NAFLD include insulin resistance, metabolic disorders, and inflammation [185]. NAFLD independently increases the risk of arrhythmia recurrence following AF ablation [188]. Additionally, advanced liver fibrosis is linked to a higher incidence of AF in individuals with NAFLD [188].

When CLD and AF coexist, a greater risk of stroke compared to those without AF has been reported, correlating with the severity of CLD and increased mortality rates. Moreover, AF is frequently observed among individuals with liver cirrhosis (LC), with an incidence of 11.6%, which rises in those with decompensated LCD, alcoholic liver disease, NASH, elderly, and those with comorbidities [189]. A 1.44 times higher risk of mortality has been described in these patients [190]. 

Managing OACs therapy in patients with AF and CLD presents significant challenges. Additionally, there are limited data on the use of DOACs in CLD patients, as they are underrepresented in DOACs clinical trials. This issue arises because individuals with LD have traditionally been considered at a higher risk of bleeding. Moreover, DOACs are partially or entirely metabolized by liver cells, and CLD may influence drug clearance and metabolism. Furthermore, patients with CLD were mostly excluded from the CRT on DOACs.

The metabolism of DOACs, such as apixaban, rivaroxaban, and edoxaban, is significantly affected by liver function, with hepatic metabolism rates of 75%, 65%, and 50%, respectively. In contrast, dabigatran etexilate, a prodrug, is not influenced by hepatic metabolism.

## 9. Chronic Kidney Disease (CKD)

CKD has been shown to be an independent risk factor for the increased incidence and prevalence of AF.

AF and chronic kidney disease (CKD) are interconnected conditions with shared risk factors, such as AH, DM, and CAD. 

**Figure 7:**
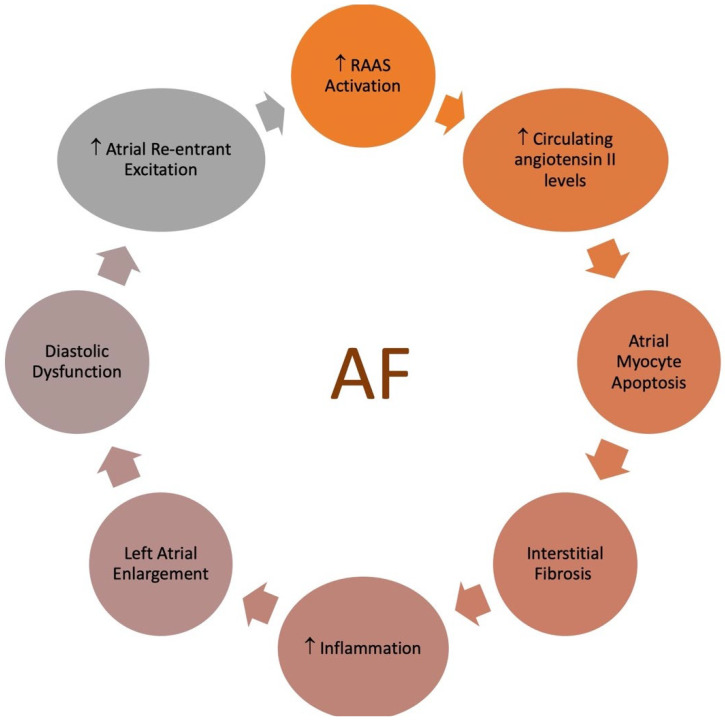
Relationship between chronic kidney disease and atrial fibrillation.

As the prevalence of both AF and CKD continues to rise, a growing number of patients are affected by these concurrent conditions. Thus, understanding the implications of coexisting AF and CKD is crucial (Figure 7).

CKD activates the renin–angiotensin–aldosterone system (RAAS) and the sympathetic nervous system, leading to oxidative stress, systemic inflammation, and volume overload. Angiotensin II, a key component of the RAAS, can increase atrial pressure, accelerate atrial fibrosis, and cause ion channel dysfunction. These effects contribute to structural and electrical remodeling of the atria, ultimately resulting in AF. CKD patients typically show elevated levels of inflammatory markers, such as C-reactive protein (CRP), interleukin-6 (IL-6), fibrinogen, and other molecules. These inflammatory markers increase as the disease progresses [191]. Chronic systemic inflammation can lead to fibrosis, hypertrophy, and cellular apoptosis in the atria. Cytokines and growth factors associated with systemic inflammation activate cardiac fibroblasts, which then contribute to the development of fibrosis.

A significant increase in thromboembolic risk has been described [191] in patients with both AF and CKD. The initiation of OACs presents a significant clinical challenge in these patients. In CKD, various pathophysiological factors contribute to the risk of ischemic stroke and bleeding as renal function deteriorates, regardless of OACs.

The current evidence indicates that DOACs are generally preferred over VKAs due to their potentially greater safety and efficacy in CKD, as well as a lower risk of vascular calcification and anticoagulant-related nephropathy.

Despite the limited data on efficacy and safety outcomes, both the FDA and the EMA have approved reduced doses of apixaban, edoxaban, and rivaroxaban for patients with an estimated glomerular filtration rate (eGFR) of 15–30 mL/min. Additionally, the FDA has approved a specific low dose of dabigatran (75 mg twice daily) for these patients, according to pharmacokinetic data. Until RCTs specifically address optimal management, clinical decisions should be guided by the available limited data, emphasizing individualized treatment plans and collaborative discussions between physicians and patients.

## 10. Cancer and Hematological Disorders

AF has been observed to occur more frequently in patients with malignancies. The incidence of AF in cancer patients could be attributed to coexisting medical conditions, direct effects of cancer, or complications arising from cancer treatments, such as surgery, chemotherapy, or radiation therapy [192]. Furthermore, the relationship between AF and cancer may be explained by shared risk factors or systemic disease mechanisms, including aging, CV and metabolic disorders, cancer invasion, inflammation, and hypoxia. Inflammation might serve as a common underlying factor linking both conditions [192,193]. Importantly, a high prevalence of CV risk factors and disease has been reported among patients with cancer and AF. Conversely, AF significantly elevates the risk of CV complications, influencing the prognosis of malignant diseases and also representing a substantial challenge in the therapeutic management of cancer patients [194]. 

Even though the CHA2DS2-VASc score and HAS-BLED score have not been thoroughly validated in cancer patients, these tools should be utilized for diagnosing and managing AF in cancer patients [3].

Importantly, anticoagulation strategy is largely underused in cancer patients with AF, especially before cardiology consultation, regardless of the CHA2DS2VASc and HAS-BLED score values. However, as has been reported in the Blitz AF Cancer, following the MDTs’ evaluation, including cardiological evaluation, the majority of patients undergo an appropriate anticoagulation therapy [194]. VKAs are less likely to be prescribed for cancer patients due to their limitations and potential drug interactions in this context. Additionally, low-molecular-weight heparins (LMWHs) have not been shown to prevent stroke or systemic embolism in AF effectively. Their use may only be warranted based on their established effectiveness and safety in managing venous thromboembolism (VTE).

There has been no dedicated randomized controlled trial investigating the use of DOACs specifically for AF in cancer patients [21,195]. However, extensive observational studies and post hoc analyses of pivotal trials involving DOACs in AF suggest that DOACs are associated with lower risks and are at least as effective as VKAs in individuals with AF who also have active cancer [21,195] (Table 2).

In healthy patients, CA is a well-researched, safe therapeutic option; however, the literature on the safety of CA for AF in cancer patients is scarce and restricted to a few centers. In selected cancer patients with symptomatic paroxysmal AF who have not responded to ADDs, the possibility of CA should be addressed, taking into account cancer status prognosis, patient preferences, the presence of structural heart disease, and individual bleeding and thrombotic risks within a MDT strategy [193].

In light of the rapidly changing clinical landscape, patients with active cancer stand to gain from a more intensive follow-up regimen involving frequent re-evaluations. Determining the optimal anticoagulation strategy for cancer patients necessitates a multidisciplinary approach that considers individualized factors, such as bleeding and thrombotic risks, potential drug interactions, patient preferences, and regular clinical assessments [193].

## 11. Atrial Fibrillation in Pregnancy

AF is a commonly encountered condition during pregnancy, often leading to combined incidences due to the adoption of similar management strategies. However, there is a dearth of specific data regarding the prevalence of atrial flutter as an independent entity. Within a cohort study comprising 1321 pregnant women diagnosed with congenital, valvular, and CAD, it was found that 1.3% of the women developed either AF or atrial flutter [196]. This occurrence was predominantly noted during the second trimester, with a heightened frequency observed among those with mitral valve pathology. Notably, depending on the severity and nature of the underlying structural heart ailment, incidences could escalate to as high as 39.2% [196].

In hemodynamically stable patients, the initial management typically involves rate control, although direct current cardioversion (DCCV) may be favored for the index episode or in cases of inadequate rate control. DCCV should be performed within 48 h of AF onset to minimize stroke risk, with the potential necessity for transesophageal echocardiography if the onset of AF is uncertain [196]. Thromboembolic risk management is essential, with preference given to heparin compounds, especially low-molecular-weight heparin [196].

Pregnancy increases the risk of thrombosis, although the data on stroke risk associated with AF and atrial flutter during pregnancy are limited. Full anticoagulation is recommended for patients with mitral stenosis [197]. However, the CHA2DS2-VASc score has not been validated for use during pregnancy [196]. Therefore, the 2018 ESC guidelines suggest employing the same criteria for stroke risk stratification as in non-pregnant patients [197].

**Figure 8:**
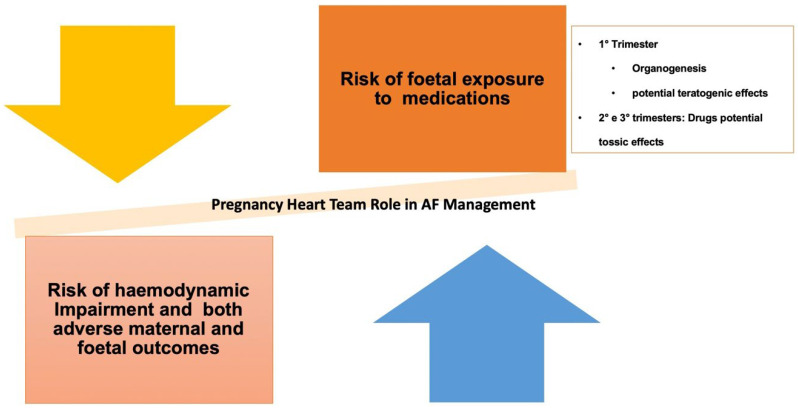
Pregnancy heart team (PHT) involvement in AF patient management. PHT aims to provide the quality of care for pregnant women with complex medical conditions. This team would comprise various specialists, including cardiologists, gynecologists, anesthesiologists, as well as other experts, such as geneticists, neonatologists, cardiac surgeons, endocrinologists, and oncologists. The PHT aims to provide comprehensive care, from pre-conception counseling to postpartum follow-up, including monitoring during pregnancy and delivery.

Pregnancy care should be overseen by a specialized pregnancy heart team (PHT) or cardio-obstetric team comprising experienced cardiologists, gynecologists, anesthesiologists, obstetricians, and nurses [198,199] (Figure 8). It is recommended to have at least one consultation per trimester to ensure the safety of both the mother and the fetus [200]. Timely interventions may be necessary to maintain optimal fetal well-being, irrespective of pre-existing heart conditions. Decision making should be guided by the gestation stage, carefully considering the potential teratogenic effects of medications [200]. The primary goals of the PHT include comprehensive counseling, meticulous planning of delivery timing and method, and diligent postpartum monitoring [198,199,200].

## 12. Technological Tools and E-Health (Remote Monitoring and Wearable Tools)

The detection of AF can be a challenge due to its paroxysmal and often asymptomatic nature [201]. However, an early diagnosis is associated with a reduction in morbidity or mortality and should be our target for AF treatment. Continuous monitoring represents a good strategy to increase the possibility of diagnosis [202]. In addition to CV implantable electronic devices (CIEDs), wearable devices such as self-applied ECGs [203] and smartwatches [204] enabling continuous heart rate monitoring have been developed.

These devices may be utilized to monitor patients with established asymptomatic AF to evaluate the rate and burden of AF, and they may also assist in identifying individuals with undiagnosed AF.

The introduction of digital health technology may contribute to a more structured multidisciplinary approach, and the optimization of therapeutic strategies has recently enhanced AF management [205], supporting a patient-focused approach and decision making involving several specialists, general doctors, patients, specialists, caregivers, and nurses.

In this sense, novel technological equipment covers all ends of the spectrum, from mobile health (mHealth) to communication tools [206], resulting in earlier AF detection, which leads to improved drug adherence [207], a more appropriate OAC use [208], and better management of comorbidities and CV risk factors [208,209,210,211,212,213,214,215].

### Wearables

Wearable technologies, simply termed “wearables”, were developed only recently and defined as any electronic device that can be worn, put on, removed from the body, or worn into clothing or as accessories [216]. A wide range of smart wearables, including smartwatches, chest patches/straps, or sensors integrated into clothing and footwear, can continuously identify AF using automated algorithms [217,218] and offer individualized and self-managed patient care with a positive impact on patient–physician relations.

Depending on the wearable devices, the algorithm’s accuracy in detecting AF varies from 70 to 90% [219]. The most used wearable technology is photoplethysmography, which obtains information about the heart rhythm through blood volume changes in a peripheral vascular bed that is synchronous with the heartbeat. Other technologies include electrocardiogram, echocardiography, or phonocardiogram, which detects subaudible vibrations created by opening and closing heart valves.

**Figure 9:**
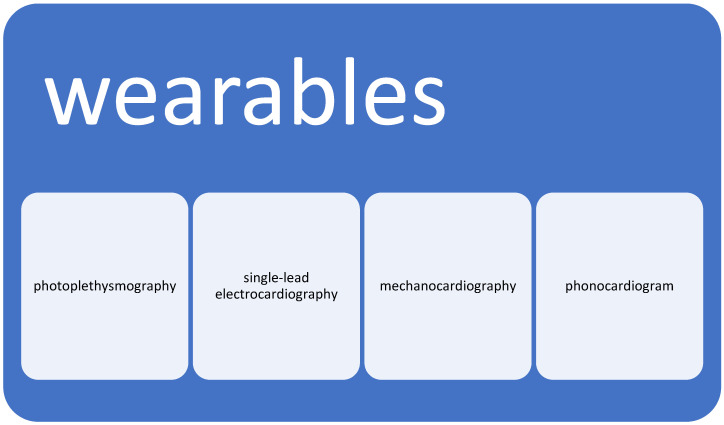
Technologies of the wearables.

These devices demonstrated excellent diagnostic accuracy in detecting AF, as indicated by the high sensitivity and specificity (Figure 9). Both photoplethysmography and electrocardiography exhibit comparable effectiveness in identifying arrhythmias [220].

Regarding the clinical impact, wearables demonstrated a higher detection rate of arrhythmias. In a comprehensive smartwatch evaluation designed to detect AF, irregular pulses were identified in 0.52% of the patients.

In this cohort, the positive predictive value (PPV) for identifying AF on an electrocardiogram (ECG) concurrently with a subsequent irregular pulse notification was 0.84. Similarly, the PPV for identifying AF on ECG simultaneously with a subsequent irregular tachogram was 0.71 [204].

On the contrary, a recent meta-analysis did not report a significant decrease in stroke incidence. However, it is worth noting that this analysis encompassed a study by Halcox et al., which had a relatively small sample size and thus limited statistical power. Despite this, the utilization of wearables has demonstrated a positive economic impact. Screening asymptomatic patients with a consequent reduction in stroke occurrences not only enhances the quality of life but also proves to be cost effective [221].

Wearables have been defined as “novel tools that add to the diagnostic possibilities in patients with a high risk of AF” but underline that appropriate management pathways are still incomplete [3,215].

## 13. Conclusions

AF necessitates a comprehensive, all-encompassing, and collaborative approach to patient management, wherein patients actively engage in partnership with healthcare providers. Simplifying the process of caring for AF patients in everyday clinical settings is a demanding yet indispensable aspect of effective AF management. Over recent years, significant advancements have been achieved in AF detection and management, with the timely incorporation of new evidence being a key feature of the third edition of the ESC guidelines on AF.

Ensuring optimal treatment for AF patients demands a robust healthcare infrastructure and substantial financial investment. The allocation of resources will naturally differ depending on the structure of healthcare systems and budget limitations in various regions. The ESC Atlas on Cardiovascular Disease has highlighted stark disparities in access to resources related to AF management. Therefore, it is crucial to strategize the efficient utilization of existing resources to mitigate stroke risk, alleviate symptoms, and address comorbidities.

## Figures and Tables

**Table 1:** Studies on atrial fibrillation better care (ABC) pathway.

Author	Patients	N° of ABC Patients	N° of Non-ABC Patients (%)	Median Follow-Up	Results of ABC vs. Non-ABC
Romiti GF. Post hoc analysis GLORIA-AF Registry [11]	24,608	23.901: −5285 (21.5%) adherent to 1 criterion; −12,112 (49.2%) adherent to 2 criteria; −6504 (26.4%) adherent to 3 criteria	707 (2.9%)	1 year	-↓Risk for the primary outcome (*p* < 0.0001) -↓Risk of mortality (*p* = 0.048) -↓Thromboembolism (*p* = 0.0078) MACE (*p* = 0.0071)
Yon M. et al. [12]	204,842	10,129 (32.0%)	66,778 (38.6%)	6.2 ± 3.5 years	-↓All-cause death (*p* < 0.001) -↓Composite outcome (death, ischemic stroke, MB, and AMI) (*p* < 0.001)
Prietti M. Post hoc analysis AFFIRM [13]	3169	222 (7%)	2947(93%)	3.7 years	-↓All study outcomes (*p* < 0.001) -↓Risk of all-cause death -↓Composite outcome -↓First hospitalization
Domek M. et al. Gulf SAFE registry [14]	603	86 (14.2)	517 (85.8)	1 year	-↓Mortality (*p* = 0.0014) -↓Risk of all-cause death -↓Risk of composite outcome after 6 months and at 1 year
Gumprecht J. [15]	2021	168 (8.3)	1853 (91.7)	1 year	↓Composite outcome (*p* = 0.02) ↓Mortality (*p* = 0.033).
BALKAN-AF survey [16]	2712	1013 (43.8%)	1299 (56.2%)		Factors which increase ABC adherence: capital city (*p* = 0.02), treatment through cardiologist’s care (*p* = 0.01), AH (*p* < 0.001), DM (*p* = 0.01), and comorbidity (*p* < 0.001). Factors which decrease ABC adherence: age ≥ 80 (*p* < 0.001), previous MB (*p*= 0.001).
Yang PS. [17]	262,987	49,533 (15%)	213,454 (85%)	5.9	-↓All-cause death (*p* < 0.001) -↓Beneficial effect on mortality in the high-frailty group (*p* < 0.001) -No statistical interaction between frailty and the composite outcome (*p* = 0.063)
Guo Y. et al. [18]	3324	1646 (49%) mAFA + ABC	1678 (51%)	262 days (ABC)/291 days non-ABC	-↓Risk of rehospitalization (*p* < 0.001) -↓Risk of clinical adverse events (*p* < 0.001)
Proietti M. [19]	6646	1996 (30.0%)	4650 (70%)	1 year	↓Rate of TE/ACS/CV death and all-cause death (*p* < 0.0001)

Abb: MACE: major adverse cardiovascular events; AH: arterial hypertension; DM: diabetes mellitus; MB: major bleeding; AMI: acute myocardial infarction; mAFA + ABC: mobile AF application incorporating the ABC; TE: thromboembolic event; ACS: acute coronary syndrome; CV: cardiovascular. ↓: reduction

**Table 2:** Evidence on the use of DOAC for AF management in cancer patients.

Trial	Type of Evidence	Number of Patients	Drug	Summary of Evidence
ROCKET-AF [153]	Sub-group analysis of RCT	640	Rivaroxaban	No efficacy and safety differences. Increased risk of bleeding
ARISTOTLE [148]	Sub-group analysis of RCT	1236	Apixaban	Similar efficacy in preventing stroke and systemic embolism. No increase in major bleeding
ENGAGE AF- TIMI 48 [125]	Sub-group analysis of RCT	1153	Edoxaban	Similar efficacy and safety
Savant AC. et al. [150]	Retrospective administrative analysis	196,521	Various DOACs	Better safety profile than warfarin
Shah S. et al. [151]	Retrospective administrative analysis	16,096	Various DOACs	Lower or similar rates of bleeding and stroke and a lower rate of incident VTE
Potter AS. et al. [152]	Retrospective single-center analysis	1133	Various DOACs	Similar risks of cerebrovascular accident, gastrointestinal bleeding, and intracranial hemorrhage
Mariani MV. et al. [154]	Meta-analysis	46,424	Various DOACs	DOACs associated with reduction in thromboembolic events and major bleeding

RCT: randomized controlled trial; DOACs: direct oral anticoagulants; VTE: venous thromboembolism.

## Data Availability

Data sharing is not applicable to this article, as no new data were created or analyzed in this study.
